# Acute cerebral infarction in a patient with an epidural catheter after left upper lobectomy: a case report

**DOI:** 10.1186/s12871-019-0695-9

**Published:** 2019-02-23

**Authors:** Asuka Kitajima, Yuji Otsuka, Alan Kawarai Lefor, Masamitsu Sanui

**Affiliations:** 1 Department of Anesthesiology and Critical Care Medicine, Jichi Medical University Saitama Medical Center, 1-847 Amanumacho, Omiyaku, Saitama 330-8503 Japan; 2 Department of Surgery, Jichi Medical University, 1-3311 Yakusiji, Shimono, Tochigi, Saitama 329-0498 Japan

**Keywords:** Epidural anesthesia, Postoperative complication, Cerebral infarction, Thoracic surgery

## Abstract

**Background:**

There are several recent reports that left upper lobe lung resection is a risk factor for the development of postoperative thromboembolism. Although administering epidural analgesia is common in thoracic surgery, anesthesiologists should be alert when administering epidural analgesia to a patient undergoing left upper lobectomy, considering the increased risk of postoperative thromboembolism and the potential need for anticoagulation or fibrinolytic therapy in the immediate postoperative period.

**Case presentation:**

A seventy-one-year-old female with a metastatic lung lesion developed a cerebral infarction approximately 30 h after video-assisted thoracoscopic left upper lobectomy. Cerebral intravascular therapy was indicated and the epidural catheter was removed immediately to avoid formation of an epidural hematoma. Approximately four hours after onset, reperfusion was successfully established by aspiration of endovascular thrombi. She recovered with mild residual paralysis of the left upper extremity and was transferred to a rehabilitation facility.

**Conclusions:**

We present a patient with a cerebral infarction after left upper lobectomy. Left upper lobectomy is associated with an increased risk of postoperative thromboembolism. Although the exact mechanism of thrombosis after left upper lobectomy is unclear, a judicious decision should be made regarding epidural catheter placement for postoperative analgesia.

## Background

Paraplegia due to acute spinal epidural hematoma after the placement or removal of epidural catheters is very rare, but once a hematoma develops, the prognosis is often devastating. Known risk factors for acute spinal epidural hematomas include thrombocytopenia, anticoagulant or antiplatelet therapy, and being an elderly female.

There are several recent reports that left upper lobe (LUL) lobectomy is a risk factor for the development of postoperative cerebral thromboembolism [1–3]. The mechanism of cerebral infarction after LUL lobectomy may include stasis of blood flow in the stump of the left upper pulmonary vein because the left upper pulmonary vein stump may be longer than other pulmonary vein remnants for anatomical reasons [2]. Although administering epidural analgesia is common in thoracic surgery, anesthesiologists should be alert when administering epidural anesthesia to a patient undergoing LUL lobectomy, considering the risk of postoperative cerebral thromboembolism and potential need for anticoagulation or fibrinolysis therapy in the immediate postoperative period. We present a patient who underwent LUL lobectomy, complicated by the development of a postoperative cerebral thromboembolism. The epidural catheter was immediately removed to allow urgent administration of intravascular therapy. A neuraxial hematoma did not develop.

## Case presentation

A 71-year-old female with a pulmonary metastasis from primary sigmoid colon cancer presented for video-assisted thoracoscopic LUL lobectomy. She underwent resection of sigmoid colon cancer (StageIIA T3N0M0) five years previously. She did not receive adjuvant chemotherapy. Recent computed tomography scan revealed a mass in the left upper lobe of the lung, and she was admitted to undergo video-assisted thoracoscopic LUL lobectomy. She had a history of hypertension and osteoporosis, treated with raloxifen, alfacalcidol, fexofenadine hydrochloride, pseudoephedrine and esomeprazole magnesium hydrate. She smoked two packs of cigarettes per day for 45 years. Physical examination on admission was unremarkable. Preoperative electrocardiogram showed sinus rhythm with an incomplete right bundle branch block. After placement of an epidural catheter between the fifth and sixth vertebrae, general anesthesia was induced with remifentanil and propofol. Tracheal intubation was accomplished using rocuronium bromide. Combined epidural and general anesthesia was maintained with remifentanil, desflurane and ropivacaine. The LUL lobectomy proceeded without difficulty with an operating time of 157 min. No arrhythmias or severe hypotension were detected during the surgery. She was transferred to a general ward after extubation in the operating room.

**Fig. 1:**
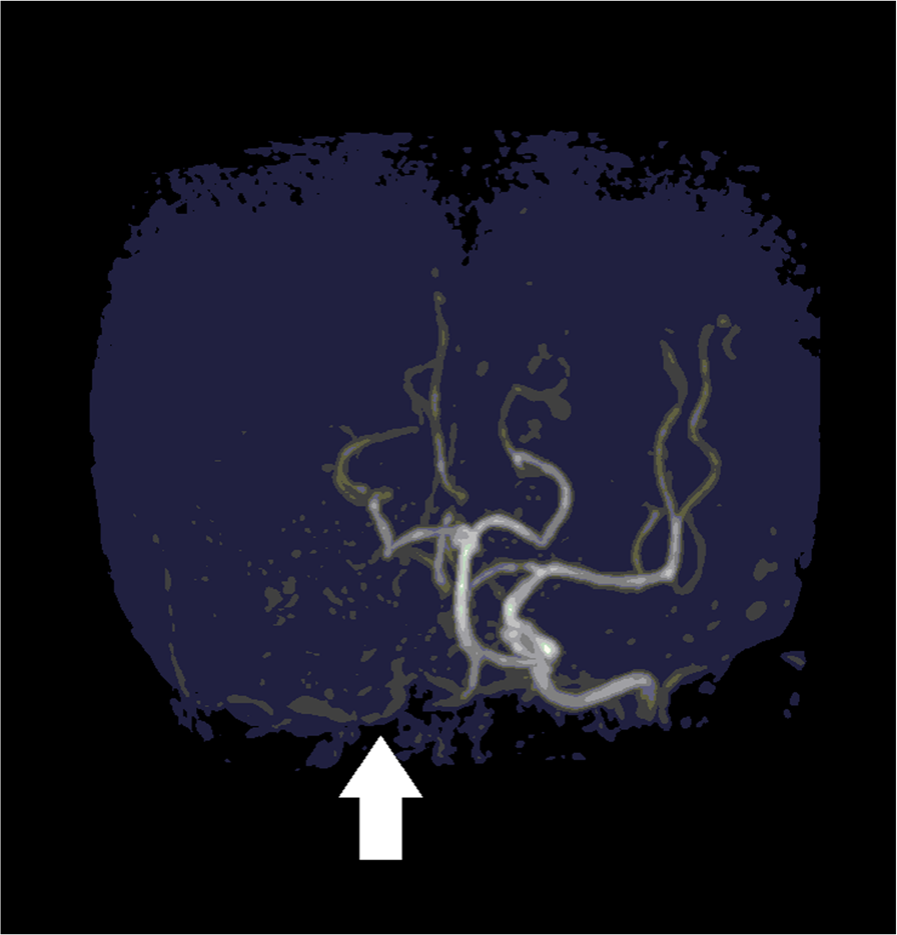
Cerebral magnetic resonance angiography. Cerebral magnetic resonance angiography performed 30 h after left upper lobe resection demonstrates interruption of blood flow in the right internal carotid artery

The postoperative course was uncomplicated with no episodes of atrial fibrillation on the first postoperative day. On the evening of the second postoperative day, she was seen to lean suddenly to the left after urinating. She developed left hemiparesis, right conjugate deviation and dysarthria. She underwent emergency magnetic resonance imaging after immediate removal of the epidural catheter. Cerebral magnetic resonance angiography revealed cessation of blood flow in the right internal carotid artery (Fig. 1). An acute cerebral infarction was diagnosed and she was transferred to another hospital to receive intravascular therapy. Initially, 4000 units of heparin were given intravenously. Four hours after onset of arterial occlusion, extensive dark brown thrombi were removed though the intravascular catheter, and cerebral perfusion was reestablished. She received protamine at the end of procedure. Anticoagulation therapy was delayed until postoperative day 21 because a minimal hemorrhagic infarction developed. She recovered with only mild paralysis of the left upper extremity and was transferred to a rehabilitation facility on postoperative day 37.

## Discussion

The incidence of cerebral infarction after pulmonary lobectomy or segmentectomy is reported to be just 0.6% [4]. This incidence is similar to that after other types of operations such as general surgery, cardiac surgery, etc. However, it has been recently reported that the incidence of cerebral infarction following LUL lobectomy is as high as 4.5% [1]. The mechanism of cerebral infarction after LUL lobectomy may include stasis of blood flow in the stump of the left upper pulmonary vein [2]. The left upper pulmonary vein stump may be longer than other pulmonary vein remnants for anatomical reasons [2]. Theoretically, a thrombus may easily be generated in a long vein remnant due to stasis [3]. In the present patient, a potential embolic source may have been in the left upper pulmonary vein remnant since the patient had no features consistent with perioperative atrial fibrillation, or a plaque in the aorta or carotid arteries. The thrombi retrieved by intravascular aspiration appeared relatively fresh, suggesting that they were formed during or immediately after surgery. For these reasons, the left upper pulmonary vein stump is a likely source of the emboli. Therefore, early postoperative anticoagulation may be indicated to prevent thrombus formation.

In the present patient, we removed the epidural catheter immediately after recognizing the cerebral infarction to maximize the time from removal until initiating the planned intravascular treatment for the cerebral embolism. Approximately four hours after removing the epidural catheter, intravascular thrombus aspiration was performed. Although 4000 units of heparin were administered intravenously at the beginning of the intravascular procedure, no thrombolytic medication was used. A four-hour interval between epidural catheter removal and administration of intravenous heparin may be sufficient. The American Society of Regional Anesthesia and Pain Medicine guideline states that a one-hour interval between needle placement and intravenous heparin administration decreases the risk of significant bleeding [5]. Early detection and selective intravascular therapy may have contributed to the good outcome.

Cerebral infarction can occur in patients undergoing LUL lobectomy even when perioperative intravenous heparinization is performed [1, 3]. To prevent thrombus formation, prophylactic subcutaneous heparin injection is probably insufficient and therapeutic systemic heparinization should be considered postoperatively. However, it is not recommended to remove the epidural catheter in patients undergoing continuous intravenous heparinization, since that may increase the risk of epidural hematoma formation. An epidural catheter can be kept in place while a patient is undergoing anticoagulation therapy as long as the acute pain service maintains close observation of the patient. However, migration or spontaneous removal of the catheter can happen. Clinically significant movement of catheters was reported in approximately 30% of patients with postoperative epidural catheters [6, 7], while accidental removal was reported in approximately 17% of catheters [8]. Fibrinolytic therapy may be needed in specific situations. Although this patient was expeditiously given intravascular therapy, this intervention is not ubiquitously available. In facilities where intravascular therapy is not available, it is reasonable to perform fibrinolytic therapy in patients with postoperative cerebral infarction [9]. Nevertheless, the safety of epidural analgesia for patients who have the potential to receive fibrinolytic therapy has been debated. It is reasonable to use other treatment modalities other than epidural analgesia because a patient who underwent LUL lobectomy may develop thromboembolism with increased likelihood and may need early postoperative anticoagulation or fibrinolytic therapy.

In our institution, we have implemented routine intravenous systemic heparinization for three days in patients following LUL lobectomies after having the experience of treating this patient. Along with this protocol for postoperative anticoagulation therapy, we changed postoperative analgesia from the routine placement of epidural catheters to intercostal nerve blocks with intravenous patient-controlled analgesia. After these policy changes, approximately one hundred LUL lobectomies have been performed, and no postoperative thromboembolic events have occurred. In contrast to previous reports [1, 3], this experience suggests that early postoperative intravenous systemic heparinization may be effective to prevent postoperative thromboembolism, although a randomized controlled study with longer follow-up and a larger sample size is necessary to determine the efficacy, safety and optimal duration of this practice.

## Conclusions

We present a patient who developed a cerebral infarction after a LUL lobectomy. Although the exact mechanism of thrombosis after LUL lobectomy has been not elucidated, a judicious decision should be made regarding epidural catheter placement for postoperative analgesia. In the future, prophylactic anticoagulation may be indicated depending on the specific type of pulmonary resection.

## Abbreviations

LUL: left upper lobe
